# ACE configurator for ELISpot: optimizing combinatorial design of pooled ELISpot assays with an epitope similarity model

**DOI:** 10.1093/bib/bbad495

**Published:** 2024-01-04

**Authors:** Jin Seok Lee, Dhuvarakesh Karthikeyan, Misha Fini, Benjamin G Vincent, Alex Rubinsteyn

**Affiliations:** Lineberger Comprehensive Cancer Center, University of North Carolina at Chapel Hill, Chapel Hill, NC; Computational Medicine Program, UNC School of Medicine, Chapel Hill, NC, USA; Curriculum in Bioinformatics and Computational Biology, UNC School of Medicine, Chapel Hill, NC, USA; Lineberger Comprehensive Cancer Center, University of North Carolina at Chapel Hill, Chapel Hill, NC; Computational Medicine Program, UNC School of Medicine, Chapel Hill, NC, USA; Curriculum in Bioinformatics and Computational Biology, UNC School of Medicine, Chapel Hill, NC, USA; Lineberger Comprehensive Cancer Center, University of North Carolina at Chapel Hill, Chapel Hill, NC; Department of Microbiology and Immunology, UNC School of Medicine, Chapel Hill, NC, USA; Lineberger Comprehensive Cancer Center, University of North Carolina at Chapel Hill, Chapel Hill, NC; Division of Hematology, Department of Medicine, University of North Carolina at Chapel Hill, Chapel Hill, NC; Department of Microbiology and Immunology, UNC School of Medicine, Chapel Hill, NC, USA; Computational Medicine Program, UNC School of Medicine, Chapel Hill, NC, USA; Curriculum in Bioinformatics and Computational Biology, UNC School of Medicine, Chapel Hill, NC, USA; Lineberger Comprehensive Cancer Center, University of North Carolina at Chapel Hill, Chapel Hill, NC; Computational Medicine Program, UNC School of Medicine, Chapel Hill, NC, USA; Curriculum in Bioinformatics and Computational Biology, UNC School of Medicine, Chapel Hill, NC, USA; Department of Genetics, University of North Carolina at Chapel Hill, Chapel Hill, NC 27599 USA

**Keywords:** ELISpot, immunological assay, high-throughput assay design, assay optimization, deconvolution, protein language model

## Abstract

The enzyme-linked immunosorbent spot (ELISpot) assay is a powerful *in vitro* immunoassay that enables cost-effective quantification of antigen-specific T-cell reactivity. It is used widely in the context of cancer and infectious diseases to validate the immunogenicity of predicted epitopes. While technological advances have kept pace with the demand for increased throughput, efforts to increase scale are bottlenecked by current assay design and deconvolution methods, which have remained largely unchanged. Current methods for designing pooled ELISpot experiments offer limited flexibility of assay parameters, lack support for high-throughput scenarios and do not consider peptide identity during pool assignment. We introduce the ACE Configurator for ELISpot (ACE) to address these gaps. ACE generates optimized peptide-pool assignments from highly customizable user inputs and handles the deconvolution of positive peptides using assay readouts. In this study, we present a novel sequence-aware pooling strategy, powered by a fine-tuned ESM-2 model that groups immunologically similar peptides, reducing the number of false positives and subsequent confirmatory assays compared to existing combinatorial approaches. To validate ACE’s performance on real-world datasets, we conducted a comprehensive benchmark study, contextualizing design choices with their impact on prediction quality. Our results demonstrate ACE’s capacity to further increase precision of identified immunogenic peptides, directly optimizing experimental efficiency. ACE is freely available as an executable with a graphical user interface and command-line interfaces at https://github.com/pirl-unc/ace.

## INTRODUCTION

The adaptive immune system plays a critical role in maintaining homeostasis against pathogens and malignancies while preserving tolerance to healthy tissues [1–6]. The process of identifying the specific molecular determinants of an adaptive immune response is known as epitope mapping and is an important step for designing vaccines, developing cellular therapies and understanding the mechanisms underpinning a broad range of immunological phenomena [7–10].

The enzyme-linked immunosorbent spot (ELISpot) assay, widely used for epitope mapping, measures antigen-specific cytokine secretion, most commonly interferon-gamma (IFN-𝛾) release by T cells [9, 11–13]. Epitope mapping performed via single-peptide ELISpot demands increasing effort, reagents and biological samples with each additional peptide of interest. In contrast, pooled ELISpot combines multiple peptides in the same well, enabling immunologists to assess the immunogenicity of many candidate peptides at once [14, 15]. This increase in throughput has been instrumental in identifying immunogenic epitopes from large peptide libraries, including neoantigen predictions derived from somatic variants and the entire proteomes of human immunodeficiency virus (HIV), Middle East respiratory syndrome (MERS) and severe acute respiratory syndrome coronavirus 2 (SARS-CoV-2) [16–27].

Performing a pooled ELISpot assay requires two distinct algorithmic steps: allocating peptides to pools (design) and determining individual peptide immunogenicity from pooled experimental readouts (deconvolution). In common practice, pooled ELISpot designs either randomize peptide-pool assignment or group peptides together into equally sized pools and repeat these groups across technical replicates. A few computational methods exist that seek to optimize assay design for specific identification of immunogenic peptides using the smallest number of pools [28, 29]. These assay design approaches try to find peptide-pool assignments where pairs of peptides co-occur in pools at most once across replicates, so that the intersection of pool memberships between any two peptides is at most one. Deconvolution of such configurations is thus simplified by reverse-mapping positive pools to the set of peptides that appears once per replicate. In cases where multiple potentially immunogenic peptides can explain the pooled assay results, an additional round of ELISpot is performed to individually interrogate the peptides present in those pools [23, 30]. Recently, statistical methods have demonstrated high accuracy with single round deconvolution, directly inferring peptide-level spot counts from pool-level observations [30]. However, adoption of these deconvolution methods remains limited.

Given the non-trivial effort and computational complexity required to design and deconvolve larger experiments, surprisingly few software tools exist. One of the first tools to address this challenge, DeconvoluteThis [28], utilizes Monte Carlo methods to minimize peptide-pool overlaps in the assay design. However, the tool reports its performance under a strong prior of expected positivity rate and is no longer available on modern operating systems. In more recent years, Ström introduced a statistical framework for deconvolution and estimation of peptide spot counts from pool spot counts [30]. Strandberg has since built on Ström’s statistical deconvolution and formalized pooled ELISpot design [29] as a partially balanced incomplete block design (PBIBD) [31, 32]. To the best of our knowledge, DeconvoluteThis, Strandberg’s Shiny app and Ström’s Shiny app are the only publicly available tools that design or deconvolve pooled ELISpot assays, with only the first two tools performing these tasks end-to-end. Notably, Ström’s and Strandberg’s tools, though restrictive in choice of assay parameters, are the only publicly available and operational methods at the time of this study.

Furthermore, previous methods fundamentally overlook peptide identities when allocating peptides to pools. In practice, many experiments generate libraries where peptides share some nontrivial degree of similarity. Recent advances in protein language models such as ProtBERT [33], ProtT5 [33] and ESM [34] have demonstrated a powerful capacity to capture diverse functional features from protein sequence alone. Taken together, we hypothesized that peptide-pool assignments that consider sequence-derived functional similarities provide a new dimension to enhance ELISpot experimental efficiency, especially in reducing the total number of required pools by increasing the precision of deconvolved immunogenic peptides.

Here, we present ACE Configurator for ELISpot (ACE), a program that facilitates sequence-aware ELISpot assay design and deconvolution of immunogenic peptides end-to-end. We fine-tuned ESM-2 to predict epitope similarity and demonstrate ACE’s robustness by rigorous benchmarking on various real-world scenarios.

## RESULTS

### Motivating a sequence-aware approach to pooled ELISpot design

Existing pooled ELISpot assay design techniques seek to minimize co-occurrence of peptide pairs between pools to aid empirical deconvolution of immunogenic peptides [28, 29]. We tested the impact of co-occurrence on deconvolution accuracy and observed an inverse relationship between the number of peptide co-occurrences and precision of immunogenic peptide identification (Figure S1). Interestingly, we observed variability in the precision levels across simulations of equivalent designs with minimal co-occurrence. We found that this variability is driven by grouping of immunogenic peptides (Supplementary Note 1). We hypothesized that peptide similarity might be an additional important design consideration in pooled ELISpot assays.

### Overview of ACE

**Figure 1:**
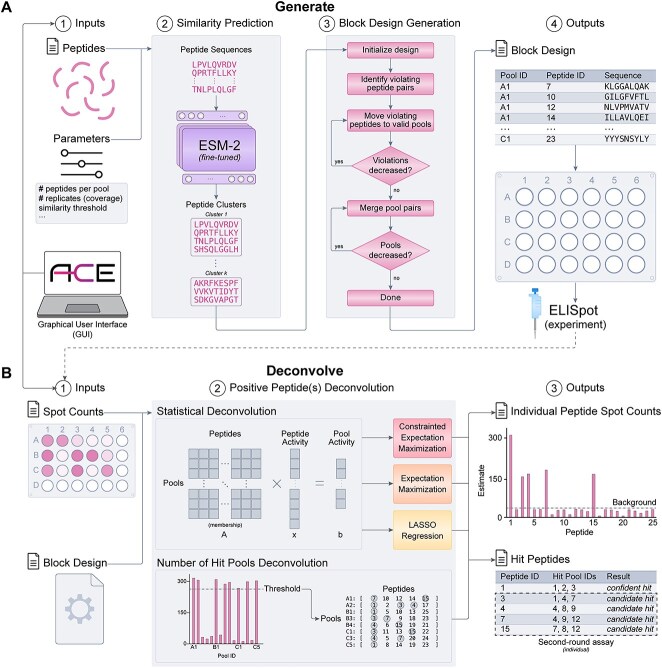
Overview of ACE. (**A**) ACE block design generation algorithm. Inputs include required assay parameters and optional sequence-specific parameters. Pairwise peptide similarity is predicted by the fine-tuned ESM-2 protein language model. Similar peptides are grouped into clusters that seed the first design replicate. Random swaps are used to reduce the number of peptide pair co-occurrence violations. The final peptide-pool assignment is mapped onto standardized microtiter plates and output as a tabular spreadsheet for ease of use at the bench. (**B**) ACE immunogenic peptide deconvolution algorithm. The deconvolution module receives ELISpot spot count data and the original ACE block design output. ACE performs both empirical and statistical deconvolution. Statistical deconvolution infers the individual peptide-level spot count while empirical deconvolution relies on a user-supplied pool spot count threshold to identify positive pools.

**Figure 2:**
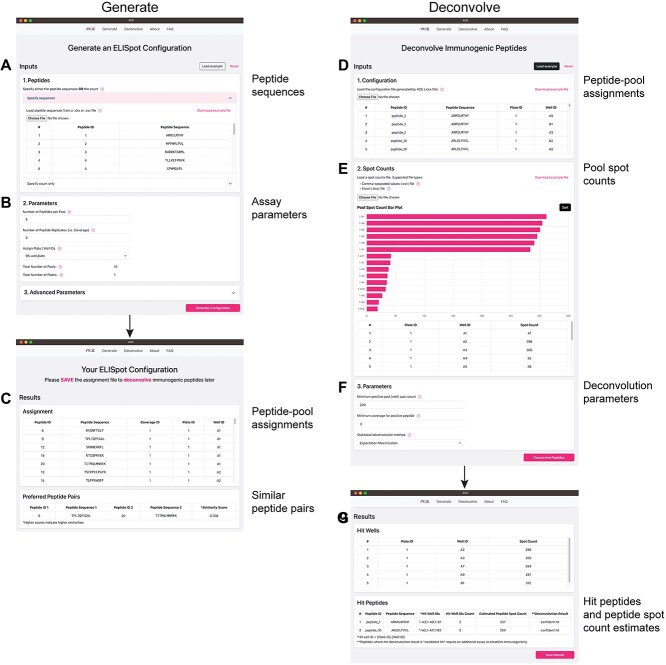
ACE GUI. The ACE *‘Generate’* UI (left panel) consists of two parts: peptide sequences and parameters. (**A**) Peptide sequences can be provided to leverage epitope similarity prediction or total peptide count can be supplied to generate sequence agnostic designs. (**B**) Assay parameters such as number of peptides per pool, replicates and plate size can be passed along with advanced sequence features as inputs. (**C**) An optimized block design is produced and available for save. The ACE ‘Deconvolve’ (right panel) consists of three parts: configuration, spot counts and parameters. (**D**) Users supply the original ACE-generated design configuration along with the experimentally derived pool spot counts. (**E**, **F**) A bar plot is dynamically generated based on the pool spot counts to help determine the positivity threshold for deconvolution. (**G**) Predicted immunogenic peptides are presented on the deconvolution results page and are available for save in a tabular format.

Building on these insights, we developed ACE, a program that supports end-to-end ELISpot experiments from peptide-pool assignment to deconvolution of immunogenic peptides (Figure 1). ACE is available as an open-source Python package and as a standalone executable with a graphical user interface (GUI) for a streamlined experience (Figure 2).

Experimentalists can provide their desired configuration parameters and generate a design that respects these constraints (Figure 2A–C). In the design step, ACE uses a fine-tuned ESM-2 model [35] to predict epitope similarity, which we define as the degree to which two peptides share cognate T-cell receptors (TCRs). ACE clusters these immunologically similar peptides, thereby optimizing for placement of potentially immunogenic peptides.

To deconvolve immunogenic peptides, experimentalists provide ACE with measured pool spot counts along with the original ACE design file. The GUI facilitates a data-driven approach to deconvolution, providing dynamic visualization of pool spot counts. This enables users to make informed decisions in setting the cutoff between positive and negative wells (Figure 2D–G).

ACE produces a comprehensive set of deconvolution results from both empirical and statistical approaches. We categorize putative immunogenic peptides into two types: confident and candidate hits. While confident hits have at least one positive pool that uniquely identifies a putative immunogenic peptide, candidate hits co-occur with other putative hits across all pools and require additional assays to confirm their immunogenicity.

ACE implements several statistical deconvolution methods for flexibility and user preference. ACE includes the expectation–maximization (EM) deconvolution method from a prior study [30] and we found no major differences in performance against their implementation (Figure S2). We also introduce two novel deconvolution heuristics. The first is a constrained expectation–maximization (CEM) technique, modeled after the Filtered Expectation–Maximization Method for ELISpot (FEMME) approach [29]. Both strategies extend the EM method to enhance the precision in peptide spot count estimation by refining the set of putative hits. Whereas FEMME excludes peptides that do not appear in any positive wells, CEM retains peptides that appear in positive wells for every replicate. The second new approach is least absolute shrinkage and selection operator (LASSO) deconvolution, which leverages LASSO regression for sparse solutions suitable for identifying a small number of immunogenic peptides in a large library.

To determine the optimal default settings for ACE, we simulated every feasible combination of ACE design and deconvolution methods. The precision and recall of immunogenic peptide identification as well as the total number of pools were determined for each combination (Figure S3, Table S2). This resulted in ACE with epitope similarity and CEM as the best combination of configuration and deconvolution methods. We refer to this combination of approaches as ACE hereafter.

### ACE outperforms publicly available design and deconvolution methods *in silico*

We next performed a benchmark study against DeconvoluteThis and Strandberg’s Shiny app. The benchmark was constructed around the experimental configurations (120 and 800 peptides each with three technical replicates) presented in the original DeconvoluteThis paper [28]. This was done to include the performance of DeconvoluteThis as part of the benchmark given that it is no longer supported. We characterized the performance of the various models in a unified simulation framework with realistic parameter choices derived from the literature (see Methods).

**Figure 3:**
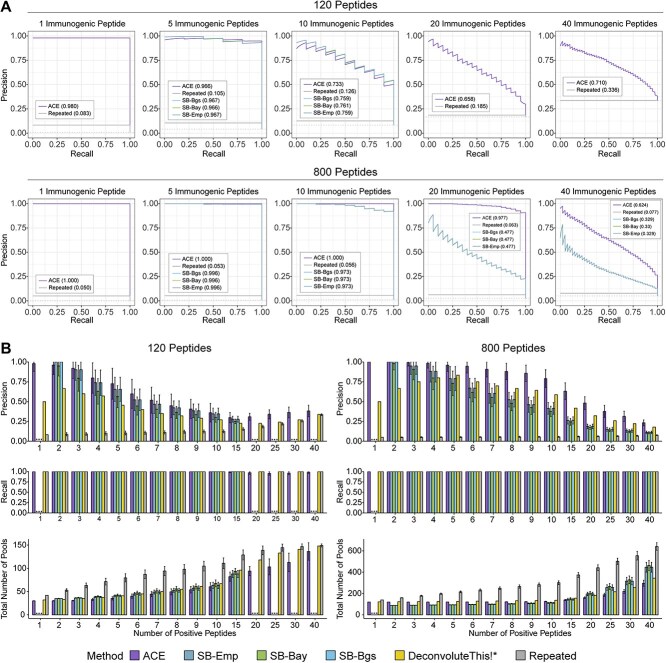
Benchmark of existing pooled ELISpot design and deconvolution methods. End-to-end design and deconvolution simulations were performed using configurations from the DeconvoluteThis paper. (**A**) Precision and recall curves for each individual design and deconvolution method are shown from simulations on a subset of representative configurations. ACE was executed with sequence similarity and constrained EM deconvolution. Repeated designs were deconvolved using empirical deconvolution (baseline). Results from Strandberg’s Shiny app all use the same matrix design and FEMME as deconvolution method and are distinguished by their positivity selection method: SB-Emp (empirical positivity selection), SB-Bay (Bayesian-predictive ELISpot criterion; BPEC), and SB-Bgs (background subtracted). (**B**) Bar plots of precision, recall and total number pools (design pools + second round pools) are shown with error bars representing $\pm 1$ SD. In (B), positions annotated with asterisk (*) indicate dropouts where fewer than 20 simulations successfully ran: 1, 20, 25, 30 and 40 positive peptides for SB-Emp, SB-Bay and SB-Bgs as well as 1 positive peptide for SB-Emp, SB-Bay and SB-Bgs.

Given the typically low incidence of immunogenic peptides across the set of possible peptides [21], we summarized the performance of these methods using precision–recall curves (Figure 3A). In the 800-peptide experiment, the average area under the precision–recall curve (AUPRC) values were 0.946, 0.799, 0.799, 0.799 and 0.058 for ACE, Strandberg (background subtracted), Strandberg (Bayesian), Strandberg (empirical) and repeated block design, respectively (Table 1). For the 120-peptide experiment, we encountered many failures with Strandberg’s three deconvolution methods and were only able to evaluate the performance of all methods on a subset of simulations. ACE and repeated design achieved an average AUPRC of 0.829 and 0.147, respectively, across all positivity rates.

**Table 1:** End-to-end ELISpot experiment benchmark results for 120 and 800 peptides

Table 1A: End-to-end ELISpot experiment benchmark results for 120 peptides							
Configuration method	Deconvolution method	Avg AUROC	Avg AUPRC	Avg Precision	Avg recall	Avg number of total pools	
ACE	Constrained EM	0.950	0.829	0.560	0.991	62	
DeconvoluteThis	DeconvoluteThis	NA	NA	0.382	1.000	73	
Random	Constrained EM	0.938	0.782	0.497	0.993	66	
Repeated	Empirical	0.715	0.147	0.147	1.000	101	
Strandberg	Background subtracted	NA	NA	NA	NA	NA	
Strandberg	Bayesian	NA	NA	NA	NA	NA	
Strandberg	Empirical (Strandberg)	NA	NA	NA	NA	NA	
NA denotes cases where method was unable to run.							
**Table 1B: End-to-end ELISpot experiment benchmark results for 800 peptides**							
**Configuration method**	**Deconvolution method**	**Avg AUROC**	**Avg AUPRC**	**Avg precision**	**Avg recall**	**Avg number of total pools**	
ACE	Constrained EM	0.996	0.946	0.757	1.000	148	
DeconvoluteThis	DeconvoluteThis	NA	NA	0.553	1.000	162	
Random	Constrained EM	0.994	0.921	0.699	0.999	155	
Repeated	Empirical	0.882	0.058	0.058	1.000	316	
Strandberg	Background subtracted	0.983	0.799	0.511	1.000	160	
Strandberg	Bayesian	0.983	0.799	0.476	1.000	164	
Strandberg	Empirical (Strandberg)	0.983	0.799	0.511	1.000	160	
NA denotes cases where the method was unable to run.							

To better understand the tradeoff between precision, recall and the number of pools used, we investigated these quantities independently (Figure 3B). All the methods were able to achieve nearly perfect recall. We found that the average precision decreased as the number of immunogenic peptides increased. Also, the number of total pools increased as the number of immunogenic peptides increased due to the need for more second round confirmatory assays. We additionally simulated and evaluated ACE under all other configurations reported in the original DeconvoluteThis paper [28]. ACE consistently outperformed DeconvoluteThis in cases of 15 or more immunogenic peptides by yielding fewer numbers of total required pools (Figure S4).

To better characterize why ACE outperformed the other methods, we next sought to analyze the distribution of estimated peptide spot counts for the simulated immunogenic and non-immunogenic peptides. We found that the ACE peptide-level spot count estimates were more robust to increasing positivity rates compared to the other methods (Figure S5).

### Ablation study

To understand the contributions of the different design and deconvolution components on overall performance, we conducted ablation studies for our methods using various intermediate versions of ACE on the 120-peptide setting (Table 2). For the design method ablation study, we fixed empirical deconvolution as the standardized deconvolution method, given its widespread use and interpretability. As expected, we observed the largest boost in performance ($\Delta AUPRC=0.280$) going from the maximally violating repeated design to the randomized block design, which has fewer peptide co-occurrence violations. When going from the randomized block design to ACE with epitope similarity turned off, we observed a modest improvement ($\Delta AUPRC=0.034$). Lastly, the inclusion of the epitope similarity module resulted in a further increase in performance ($\Delta AUPRC=0.022$).

**Table 2:** Configuration and deconvolution ablation studies

Table 2A: Configuration ablation study						
Configuration method	Deconvolution method	Avg AUROC	Avg AUPRC	Avg precision	Avg recall	Avg number of total pools
ACE	Empirical	0.855	0.484	0.484	1.000	69
ACE-0	Empirical	0.843	0.461	0.461	1.000	72
Random	Empirical	0.840	0.428	0.428	1.000	72
Repeated	Empirical	0.715	0.147	0.147	1.000	101
**Table 2B: Deconvolution ablation study**						
**Configuration method**	**Deconvolution method**	**Avg AUROC**	**Avg AUPRC**	**Avg precision**	**Avg recall**	**Avg number of total pools**
ACE	Constrained EM	0.950	0.829	0.560	0.991	62
ACE	EM	0.951	0.823	0.484	1.000	69
ACE	LASSO	0.896	0.777	0.669	0.835	44
ACE	Empirical	0.855	0.484	0.484	1.000	69

We then performed an ablation study on the different deconvolution methods using ACE with epitope sequence clustering turned on as the default design method and empirical, LASSO, EM and CEM as the different deconvolution methods. Without a continuous score for AUPRC calculation under empirical deconvolution, we artificially set this score to be the coverage of each hit peptide ranging from 0 to the number of replicates. We found that the largest contribution in deconvolution performance was going from empirical deconvolution [36] to statistical LASSO deconvolution ($\Delta AUPRC=0.294$). However, within the deconvolution space, both going from LASSO to EM ($\Delta AUPRC=0.045$) and EM to CEM ($\Delta AUPRC=0.006$) had less pronounced impacts on performance. Among the three statistical deconvolution methods, we selected CEM as the best-performing approach given its completely automated selection of positive peptide spot count thresholds following statistical deconvolution.

### Designing around epitope similarity optimizes ELISpot assays in real-world scenarios

**Figure 4:**
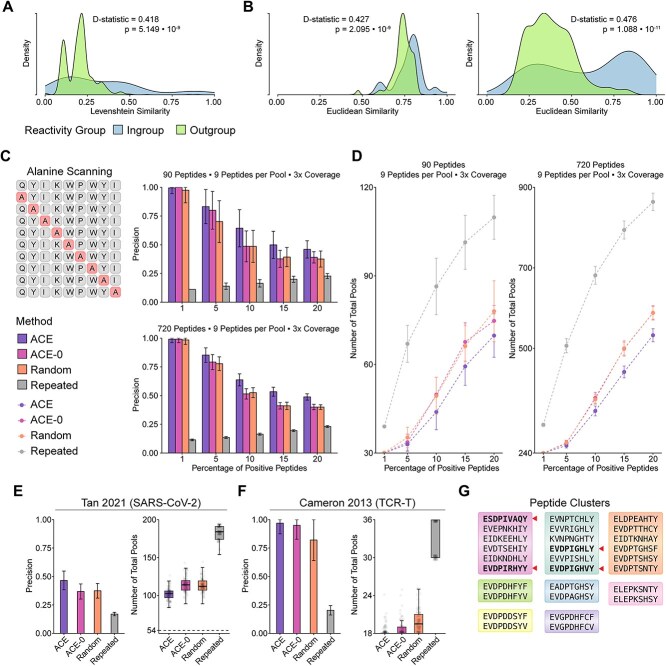
Evaluation of ACE sequence similarity prediction for efficient ELISpot assay in real-world datasets. (**A**) Distribution of pairwise sequence Levenshtein similarity scores stratified by peptides that share TCR specificity (ingroup peptides) and those with no shared TCRs (outgroup peptides). (**B**) Distribution of pairwise Euclidean distances computed on sequence embeddings from ingroup and outgroup epitopes shown for both off-the-shelf ESM-2 model and its fine-tuned counterpart. (**C**) The left panel shows a schematic for alanine scan. The right panels show average precision of simulated alanine-scanned peptide configurations by ACE with (ACE) and without sequence similarity (ACE-0), randomized block and repeated block designs across various positivity levels. (**D**) Line plots of total pools (first-round pools + candidate hit validation pools). (**E**) Evaluation of ACE sequence similarity module on SARS-CoV-2 data held-out from training ($n=177$, ${n}_{immunogenic}=37$). (**F**) Evaluation of ACE in TCR cross-reactivity setting, clustering peptides predicted to bind to MAGE A3 TCR. (**G**) Clusters from MAGE A3 experiment with immunogenic peptides bolded and highlighted with arrows. All error bars in this figure show $\pm 1$ SD.

ACE incorporates a neural engine that leverages a fine-tuned ESM-2 model to facilitate epitope similarity prediction and clustering. The model was trained on MHC-I restricted epitopes, using a contrastive loss objective designed to bring the epitopes that share a common TCR closer in the latent embedding space compared to peptides without shared TCRs. The ESM-2 8M checkpoint was used, both for its faster inference times along with its competitive validation loss compared to higher capacity checkpoints (Supplementary Note 2). Compared to the off-the-shelf ESM-2 model, the fine-tuned model assigns high pairwise similarities to peptides that bind to the same TCR even when they have high-sequence edit distances (Figure S6). Comparing the two distributions of pairwise distances using the two-sample Kolmogorov Smirnov (KS) test, the D-statistic values were 0.418, 0.427 and 0.476 for the Levenshtein similarity $\left(P=5.149\bullet{10}^{-9}\right)$, baseline ESM-2 $\left(P=2.095\bullet{10}^{-9}\right)$ and fine-tuned ESM-2 $\left(P=1.088\bullet{10}^{-11}\right),$ respectively. While all the comparisons were statistically significant, we achieved the greatest separation with ESM-2 fine-tuning (Figure 4A and B).

To evaluate the effectiveness of the peptide clustering strategy, we tested the feature using four different real-world datasets: held-out IEDB peptides [37], neoantigens from pancreatic cancer [16], immunogenic SARS-CoV-2 peptides [38, 39] and cross-reactive MAGE A3 antigens for adoptive T-cell therapy [40]. Given the frequent failure rate of Strandberg’s Shiny app and the lack of parameter selection we noted previously, we decided to compare ACE against randomized and repeated block designs for these datasets. For the comparisons of the ACE ELISpot configuration generation with and without peptide similarity prediction across a variety of real-world immunological settings, we refer to ACE without epitope similarity prediction as ACE-0 and use ACE to refer to the version with the prediction included.

Mutational scans substitute each residue of a peptide sequence and test for loss of a property of interest, such as immunogenicity. The alanine scan is a type of mutational scan where each amino acid along a protein is swapped specifically for alanine to assess critical residue positions (Figure 4C) [41]. In studies of T- and B-cell reactivity, alanine scans and other mutational scans have been used to investigate which epitope positions are significant for cognate receptor recognition [42, 43]. We benchmarked ACE’s sequence similarity prediction in the context of testing immunogenicity for alanine scanned 9-mers from the IEDB database [37]. We randomly selected 10 and 80 starting nonamers, which resulted in 90 and 720 alanine-scanned peptides. In the case of 90 alanine-scanned peptides (9 peptides per pool with 3× coverage), the average precision values across the positive peptide percentages were 0.686, 0.611, 0.586 and 0.168 for ACE, ACE-0, randomized and repeated block designs, respectively. In the case of 720 alanine-scanned peptides (9 peptides per pool with 3× coverage), the average precision values were 0.700, 0.622, 0.620 and 0.168 for the aforementioned methods. Consistent with the benchmark experiment studies so far, precision inversely correlated with the number of total pools (Figure 4D).

We next evaluated the effect of applying sequence-based clustering on ELISpot experimental efficiency on real-world datasets where immunogenicity was validated *in vitro*. The first was a set of curated SARS-CoV-2 epitopes assessed for cross-reactivity to high precursor TCRs [38]. This dataset consisted of 177 known MHC-I and MHC-II epitopes, shown to solicit CD8+ and CD4+ T-cells responses *in vitro*. Given ACE neural engine’s training on CD8+ epitopes, we performed a simulated experiment assuming that the set of 177 peptides was pulsed into an assay containing only CD8+ T cells. To fit this assumption, we considered only the 22 MHC-I restricted epitopes to be immunogenic and the 155 MHC-II epitopes to be non-immunogenic in this setting (10 peptides per pool and three technical replicates). For this dataset, the average precision values were 0.466, 0.368, 0.375 and 0.170 for ACE, ACE-0, randomized and repeated block designs, respectively (Figure 4E).

We next explored if epitope similarity-based pooling would be helpful in large protein-level epitope scans. We identified 1265 nonamers over the entire SARS-CoV-2 spike protein sequence by sliding window $\left(k=9\right)$ and found 105 (8%) matching immunogenic nonamers from the ImmuneCODE database with known TCR binding [39]. We found that ACE and ACE-0 performed similarly in this sequence set at an average of 0.496 and 0.500 precision levels, respectively, and an average recall of 1.000 for both approaches (Figure S7). We also simulated pooled ELISpot assays for 232 candidate neoantigens from a pancreatic cancer study [16] where their immunogenicity was tested via ELISpot *in vitro* (30 immunogenic). Analogous to the SARS-CoV-2 sliding window experiment, we found that ACE and ACE-0 performed similarly. The average precision levels were 0.372 and 0.381 for ACE (average recall = 0.988) and ACE-0 (average recall = 0.989), respectively (Figure S7). Although there was no significant benefit to clustering, we note that even in adversarial settings, ACE performs on par with ACE-0.

Finally, we examined ACE’s performance in the *in vitro* TCR cross-reactivity setting using a set of 37 epitopes tested for off-target T-cell response in a MAGE A3-directed adoptive T-cell therapy [40]. We used this dataset to evaluate the ability of our peptide similarity prediction module to group peptides that bind to the A3 TCR. While not perfectly grouped, we found that the partial grouping of the MAGE A3 reactive epitopes resulted in first round deconvolution precision values of 0.969, 0.950, 0.821 and 0.203 for ACE, ACE-0, randomized and repeated block designs, respectively (Figure 4F and G).

### ELISpot design space exploration

**Figure 5:**
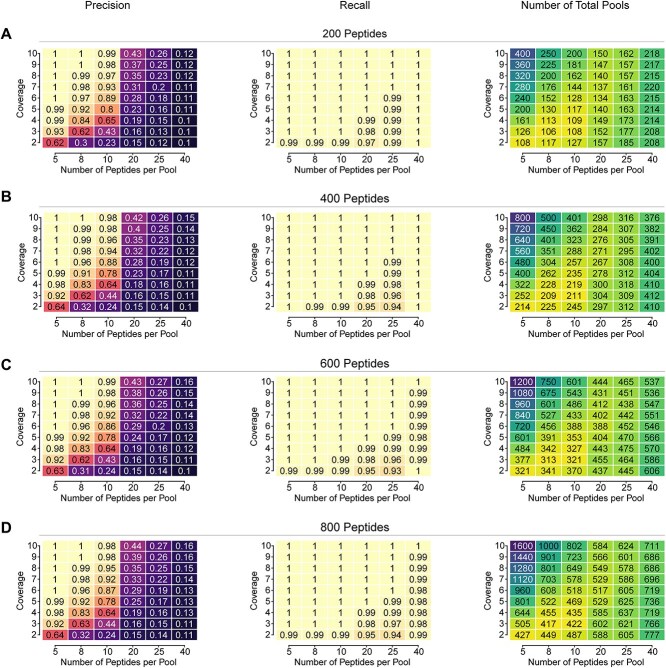
Exploration of ELISpot design optimization space. Parameter sweeps across the coverage and pool size for a fixed number of peptides were performed while measuring precision, recall and total number of pools. (**A**–**D**) 200, 400, 600 and 800 peptides were simulated for 100 runs under a 10% positivity rate, and average values are shown within the boxes.

To better inform experimentalists, we explored ACE in the context of the broader pooled ELISpot design problem by analyzing the parameter space and its impact on performance. The optimal configuration for a fixed peptide library depends on experimental constraints such as readout time, reagent usage and experimenter effort. ELISpot assay time, impacted by the number of second-round assays, is approximated by measuring first round precision. Interestingly, we found a basin of minimal experimental effort. At an assumed 10% positivity rate, the number of total pools is the smallest regardless of the number of total peptides in the experiment when eight peptides are pooled together with three technical replicates (Figure 5). Deviations from this set of parameters tradeoff the number of pools in the first round and the confirmatory second round.

### ACE runtime and peak memory usage scale linearly with assay parameters

**Figure 6:**
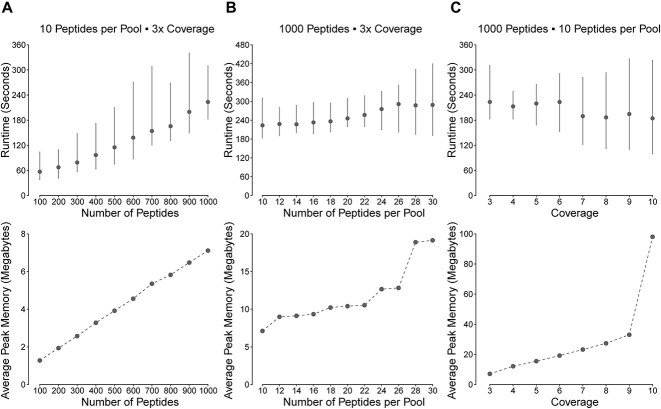
Scaling ACE. ACE ‘generate’ function peak memory usage and runtime measured across various parameter ranges with epitope similarity inference. (**A**) Runtime and peak memory usage with varying numbers of peptides while fixing 10 peptides per pool and three technical replicates. (**B**) Runtime and peak memory usage across different numbers of peptides per pool for block designs with 1000 peptides and three technical replicates. (**C**) Runtime and peak memory usage across different numbers of technical replicates for block designs with 1000 peptides and 10 peptides per pool.

To better inform users about the stability of ACE across various experiment sizes, we evaluated ACE’s scalability by analyzing runtime and peak memory usage. Given that deconvolution demonstrated faster compute times and increased consistency compared to design generation based on internal testing, we focused on ACE’s scalability across three design space axes: total peptide count, peptides per pool and coverage. Through simulations, we measured runtime and peak memory usage for ACE’s ‘generate’ function while varying these parameters. Fixing peptides per pool at 10 and coverage at three, runtime and memory showed a linear correlation with the total peptide count (Figure 6A). With a fixed peptide count of 1000 and varied peptides per pool (10–30), runtime exhibited modest fluctuations (Figure 6B). Altering technical replicates (3–10) for 1000 peptides in 10-pool settings showed linear runtime trends, but peak memory usage did not follow this pattern (Figure 6C). The peak memory usage increased linearly as a function of coverage up to 9× and increased dramatically at 10×.

## DISCUSSION

The advent of the pooled ELISpot has enabled high-throughput detection of antigen-specific T-cell responses, a fundamental step in unraveling complex immune phenomena such as cross-reactivity and immunodominance. However, current design generation and deconvolution methods lack flexibility and ignore peptide sequence information. Addressing these limitations, we developed the ACE. ACE uses advances in deep learning for a sequence-aware arbitration strategy that enhances assay efficiency and precision.

To assess ACE’s capabilities, we benchmarked against other publicly available methods, simulating realistic scenarios for ELISpot design generation and immunogenic peptide deconvolution. We validated ACE’s performance superiority over existing methods and found that its effectiveness became more pronounced as the number of positive peptides increased. As the number of positive peptides is unknown *a priori*, we expect ACE to add significant value to experimentalists in the real-world setting by enabling higher precision across a range of positivity rates. Building upon previous statistical deconvolution approaches, ACE also consistently achieved higher AUPRC values compared to the standard empirical deconvolution method. The comparison between our CEM deconvolution approach and the existing deconvolution methods further revealed that CEM peptide spot count estimation is more sensitive to higher numbers of positive peptides. Our findings further demonstrated ACE’s usefulness across diverse experimental conditions and highlighted the added benefits of sequence similarity in various settings.

ACE’s epitope similarity prediction was particularly valuable for real-world experiments, dealing with experiments involving peptide epitopes sampled from immunologically related contexts. This was demonstrated with *in vitro* validated cross-reactive MAGE A3 antigens in the context of adoptive T-cell therapy. ACE effectively clustered these cross-reactive immunogenic peptides. In studies involving MHC-I and MHC-II epitopes derived from SARS-CoV-2, ACE selectively clustered the CD8+ epitopes, boosting the precision of the assay, thereby reducing the number of total pools required.

We note that the ACE sequence-aware approach is not without limitations. We acknowledge three shortcomings related to our method. First, in some cases, ACE’s sequence-aware design heuristic did not lead to performance gains over the sequence-agnostic version of ACE. We suspect this may be due to the distribution shift of HLA alleles between training and test data, as seen in the pancreatic cancer dataset [16]. Additionally, the ACE neural engine was trained using MHC-I restricted epitopes and would not be appropriate for use with other kinds of T-cell epitopes. Third, our use of peptide similarity is an auxiliary proxy of immunogenicity. Taken together, we expect our model’s performance to increase over time as more data become available, especially with regard to the issue of HLA diversity.

In conclusion, ACE’s sequence-aware approach offers a promising avenue for improving ELISpot experimental design. By bridging the gap between sequence-level properties and assay configuration, ACE equips researchers with a powerful tool to help realize the full potential of pooled ELISpot in unraveling the complexities of adaptive immune responses. As the field of precision immunology continues to expand, exploration of the ELISpot problem space and optimization efforts become more relevant. We hope that our evaluation against other methods, coupled with the accessible implementation of empirical and statistical deconvolution in the user-friendly ACE GUI, will encourage a broader adoption. We release ACE and its accompanying analyses and recommendations to better inform pooled ELISpot experimental design, in anticipation of its increased usage.

## METHODS

### Block design generation

ACE streamlines customized pooled ELISpot assay designs for efficient immunogenic peptide deconvolution. By inputting parameters such as total peptides, peptides per pool and replicates, ACE’s ‘generate’ function outputs a valid peptide-pool mapping. When peptide sequences are provided, ACE uses its fine-tuned ESM-2 model [35] to compute pairwise similarities, respecting these pairs in the first replicate. If sequences are not supplied, ACE starts from a random peptide-pool mapping. Using a greedy random-swap heuristic, subsequent coverage peptide-pool assignments are optimized by minimizing the number of co-occurrence violations, pushing them down to further replicates. While faster methods for generating non-overlapping designs (NODs) exist that leverage orthogonality in matrix designs [29], our random-swap strategy better accommodates the epitope similarity module. The output is a user-friendly spreadsheet with peptide IDs, sequences, pool IDs and well IDs for practical bench-side use.

### Peptide similarity clustering

To boost assay precision and sensitivity, we leveraged advances in deep sequence models to pool together similar peptides predicted to bind to the same TCRs. The training data consisted of MHC-I restricted epitopes, constraining the applicability of ACE neural engine to CD8+ epitope mapping. We prioritized model accuracy as well as accessibility, selecting only those that could fit on laptop memory. ACE is packaged with the smallest ESM-2 model containing roughly 8 million parameters. This model was chosen for its inference speed (Figure S8, Table S1) and robust performance as determined in a benchmark against higher capacity ESM-2 checkpoints (35M, 150M and 650M parameters) (Supplementary Note 2). The models underwent fine-tuning with triplet loss to bring embeddings closer for peptides sharing a TCR while separating those from unrelated epitopes. In assay design generation, pairwise Euclidean distances for all peptide pairs are computed and transformed into similarity scores. Pairs above the sequence similarity threshold are filtered, retaining the two most similar peptides for each anchor to identify high-confidence similarities. Post-processing involves clustering based on the transitive property, where if peptide A is similar to B and B is similar to C, the three are clustered together. This approach proved more suitable than clustering methods such as k-nearest neighbor and agglomerative clustering, which require an unknown number of clusters during assay design.

### Simulation subroutine

We conducted benchmark studies through a unified four-step simulation framework. First, we sampled peptide sequences from reference datasets (held-out IEDB [37] and study-specific datasets [16, 38, 40]) with immunogenicity labels, repeated 100 times for each reported number of positive peptides. We sampled peptide sequences from the IEDB data held-out during ACE neural engine training using the Levenshtein distance to mimic scenarios where similar sequences are tested for immunogenicity such as neoantigens arising from somatic single-nucleotide variants (SNVs) [44]. Peptide spot counts were sampled from a generalized negative-binomial distribution, as previously described [29, 30]. We included dispersion factor as a hyperparameter, set at $\phi =1$ for default. This value corresponds to a Poisson distribution, where the parameters were ${\lambda}_{immunogenic}=300$ and ${\lambda}_{non- immunogenic}=30$. The spot count of each peptide was sampled multiple times for future pooling strategies. Simulation of false negatives were not considered due to a mechanistic gap in the field’s understanding of immunodominance [45, 46].

Second, we created pooled ELISpot designs that respected a set of specific assay parameters such as the number of peptides per pool and coverage whenever possible. We used ACE, ACE-0, Strandberg’s design method, randomized and repeated design block approaches to generate the designs. For ACE designs, we used the Euclidean distance and a similarity threshold of 0.7 with a maximum of 2000 random-swap iterations.

Third, we simulated the pool spot counts by adding peptide spots to each assigned pool. We scaled non-immunogenic peptide spot counts by dividing each count by the number of peptides in a given pool to simulate their relative background spot count contribution. We sampled three negative control wells with the same parameters for non-immunogenic peptides and assumed the same number of peptides per pool for each configuration. For Strandberg’s methods, we also sampled positive pool spot counts and generated these with the same parameters for immunogenic peptides and saturated the pool spot counts at 600. Next, we performed deconvolution of the pool spot counts using the deconvolution approaches in ACE (CEM, EM, LASSO, empirical) as well as those of Strandberg and Ström. For empirical deconvolution in ACE, we applied 3-fold the average negative control well spot counts as the minimum positive pool count [47].

We used AUPRC as a threshold independent method to evaluate each method’s ability to identify the positive and negative peptides, independent of the positivity selection criterion applied (i.e. empirical versus statistical). We considered empirical peptide positivity when peptides occurred in at least *k*-positive pools. For all simulations, *k* was set to the number of technical replicates. We also captured practical evaluation metrics such as average precision, recall and the number of total pools. For DeconvoluteThis, we calculated the precision by taking the ratio of the number of true positives to the number of predicted positives, equivalent to the number of second round pools based on the original manuscript.

### Alanine scanning experiment

We conducted *in silico* alanine scan mutagenesis as a baseline to assess sequence-informed design generation. Sequences were sampled from the held-out IEDB [37] validation set of unseen epitopes. We generated a library of wild-type and mutant peptide sequences that differed from the wild type by one residue. We assumed no change in immunogenicity status post-alanine scanning, except for mutations at anchor positions 2 and 9 [48], where non-immunogenic status was assigned if the original peptide lacked alanine at these positions. This assumption simplifies TCR binding complexity [45, 48], effectively demonstrating the benefits of clustering based on epitope similarity.

### Positive peptide identification (deconvolution)

#### Empirical deconvolution

Empirical deconvolution uses negative control wells to identify pools with an enriched spot count relative to the control wells. ACE assumes the presence of negative control wells in an ELISpot experiment and supports a number of enrichment detection methods, known as positivity selection criteria [36]. The empirical deconvolution heuristic identifies the peptides that appear in positive pools with a minimum number of occurrences (i.e. coverage). We differentiate between a ‘candidate hit’ and a ‘confident hit’ to refer to two classes of peptides that are both deemed positive. A confident hit maps uniquely to a set of pools wherein the hit is the only predicted positive in a pool for at least one replicate. A candidate hit, while also mapping to unique pools, co-occurs with other putative hit peptides across all coverages and requires an additional round of validation. We use this distinction in our calculation of an assay’s total number of pools, defined as the sum of the first round pools plus the number of candidate peptides.

#### Statistical deconvolution

In addition to the empirical deconvolution, ACE performs statistical deconvolution and estimates peptide spots to provide comprehensive results from both methods to the user (Figure 1B). The statistical deconvolution method estimates peptide-level spot counts from pool spot counts by approximating solutions to the linear system:


$$y=\beta \bullet X$$

$$y= observed\ pool\ level\ spot\ counts\in{\mathbb{R}}^i$$

$$\beta = mean\ peptide\ spot\ levels\in{\mathbb{R}}^j$$

$$X=\left[\begin{array}{ccc}{m}_{0,0}& \dots & {m}_{0,j}\\{}\vdots & \ddots & \vdots \\{}{m}_{i,0}& \dots & {m}_{i,j}\end{array}\right]$$

$${m}_{i,j}=\left\{\begin{array}{l}1,\kern0.5em peptide\ j\ was\ in\ pool\ i\\{}0,\kern0.5em otherwise\end{array}\right.$$

$${\beta}^{\ast }= mean\ peptide\ spot\ counts\ of\ constrained\ peptide\ list$$

$${X}^{\ast }= constrained\ design\ matrix$$

$${\Delta}_j={y}_j\kern0.5em -\sum_{i=1}^n{\beta}_i^{\ast }{X}^{\ast }=\mathrm{background}$$

Previously, Ström *et al*. proposed a method of using the EM algorithm for finding the maximum likelihood estimate of a set of peptide spots given incomplete data [30]. A subsequent work modified the EM approach by removing peptides that did not appear in any positive well, thereby simplifying the linear system by using the submatrices ${\beta}^{\ast }$ and ${X}^{\ast }$ [29]. Similarly, our best-performing method first identifies putative hits by empirical deconvolution and subsets the peptide-pool assignment, selecting the peptides that appear in at least *k*-positive wells for subsequent EM deconvolution. We refer to this approach as constrained EM (CEM). CEM computes the differences ${\varDelta}_j$ between the observed pool spot count and the sum of confident peptide-level spot counts for peptides in pool *j*. Based on our internal testing, we observed that the distribution of ${\varDelta}$ closely approximates the non-immunogenic peptide spot count $\lambda$ under our simulation parameters. CEM models ${\varDelta}$ as the background peptide spot count and calculates the average delta across all pools to automatically establish a peptide-level positive threshold. Furthermore, we implemented a LASSO regression model that uses the L1 loss to push coefficients to zero, a particularly useful feature for estimating sparse peptide activity when expected positivity rate is low.

**Key Points**

- We introduce peptide sequence as a novel parameter consideration for optimization of high-throughput pooled ELISpot design for use in large T-cell epitope mapping assays. To operationalize this insight, we fine-tuned ESM-2 and demonstrate its usefulness in leveraging functional similarity of MHC-I restricted epitopes from sequence alone.
- We developed ACE, a software program that designs sequence-aware pooled ELISpot configurations and deconvolves pool spot counts for identification of immunogenic peptides end-to-end. We deploy ACE as both a bench-side friendly standalone application as well as a command line interface for use on cluster nodes.
- ACE builds on statistical deconvolution methods of deriving sparse matrix solutions using expectation–maximization and we show its state-of-the-art performance.
- We performed the first systematic ablation study of both design and deconvolution of pooled ELISpot, characterizing the impact of each design and deconvolution component on performance. Additionally, we performed the first comprehensive cross-tool benchmark for pooled ELISpot design and deconvolution methods.

## Supplementary Material

ACE_Supplementary_Figures_v3_bbad495

ACE_Supplementary_Tables_v3_bbad495

ACE_Supplementary_Notes_v3_bbad495

## CODE AVAILABILITY

Both the standalone executable with a graphical user interface and the command-line interface with a Python package of ACE are freely available at https://github.com/pirl-unc/ace. The benchmark study scripts are available at https://github.com/pirl-unc/ace-analysis.

## Author Biographies

**Jin Seok Lee** is a PhD student in the Bioinformatics and Computational Biology curriculum at the University of North Carolina at Chapel Hill.

**Dhuvarakesh Karthikeyan** is a PhD student in the Bioinformatics and Computational Biology curriculum at the University of North Carolina at Chapel Hill.

**Misha Fini** is a PhD student in the Microbiology and Immunology program at the University of North Carolina at Chapel Hill.

**Benjamin Vincent** is an Associate Professor in the Department of Microbiology and Immunology and Medical Doctor in Hematology and Oncology at the University of North Carolina at Chapel Hill.

**Alex Rubinsteyn** is an Assistant Professor in the Department of Genetics and Bioinformatics and Computational Biology curriculum at the University of North Carolina at Chapel Hill.
