# Supplementary material for: ACE configurator for ELISpot: optimizing combinatorial design of pooled ELISpot assays with an epitope similarity model
Source: Brief Bioinform. 2024 Jan 4;25(1):bbad495. doi: 10.1093/bib/bbad495 (PMC10768796; doi:10.1093/bib/bbad495)
Supplement: ACE_Supplementary_Figures_v3_bbad495 [file ace_supplementary_figures_v3_bbad495.docx]

SUPPLEMENTARY FIGURES

**ACE Configurator for ELISpot (ACE): Optimizing Combinatorial
Design of Pooled ELISpot Assays with an Epitope Similarity Model**

Jin Seok Lee^1,4,5,*^, Dhuvarakesh Karthikeyan^1,4,5,*^, Misha Fini^1,3^,
Benjamin G. Vincent^1,2,3,4,5^, Alex Rubinsteyn^1,4,5,6^

^1^Lineberger Comprehensive Cancer Center, University of North Carolina at Chapel Hill, Chapel Hill, NC

^2^Division of Hematology, Department of Medicine, University of North Carolina at Chapel Hill, Chapel Hill, NC

^3^Department of Microbiology and Immunology, UNC School of Medicine, Chapel Hill, NC, USA

^4^Computational Medicine Program, UNC School of Medicine, Chapel Hill, NC, USA

^5^Curriculum in Bioinformatics and Computational Biology, UNC School of Medicine, Chapel Hill, NC, USA

^6^Department of Genetics, University of North Carolina at Chapel Hill, Chapel Hill, NC 27599 USA

*Contributed equally, co-first authors.

# Table of Contents

**Figure S1.** Characterization of the assumption behind minimizing co-occurrence violations in non-overlapping designs.

**Figure S2.** Comparison of deconvolution area under the precision-recall curves between ACE and Strom for 120 peptides.

**Figure S3.** Simulated benchmark study evaluation on 120 peptides for different combinations of design configuration and deconvolution strategies.

**Figure S4.** Comparison of ACE and DeconvoluteThis by the total number of pools.

**Figure S5.** Comparison of predicted peptide spots for 800 peptides between ACE and Strandberg deconvolution methods.

**Figure S6.** Correlation plot between Euclidean and Levenshtein similarity.

**Figure S7.** Assay performance on 1,265 SARS-CoV-2 spike protein 9-mer sequences and 232 candidate neoantigens from a pancreatic cancer cohort (Rojas et al., Nature 2023).

**Figure S8.** Speed performance benchmark on 10,000 randomly generated 9-mer sequences.


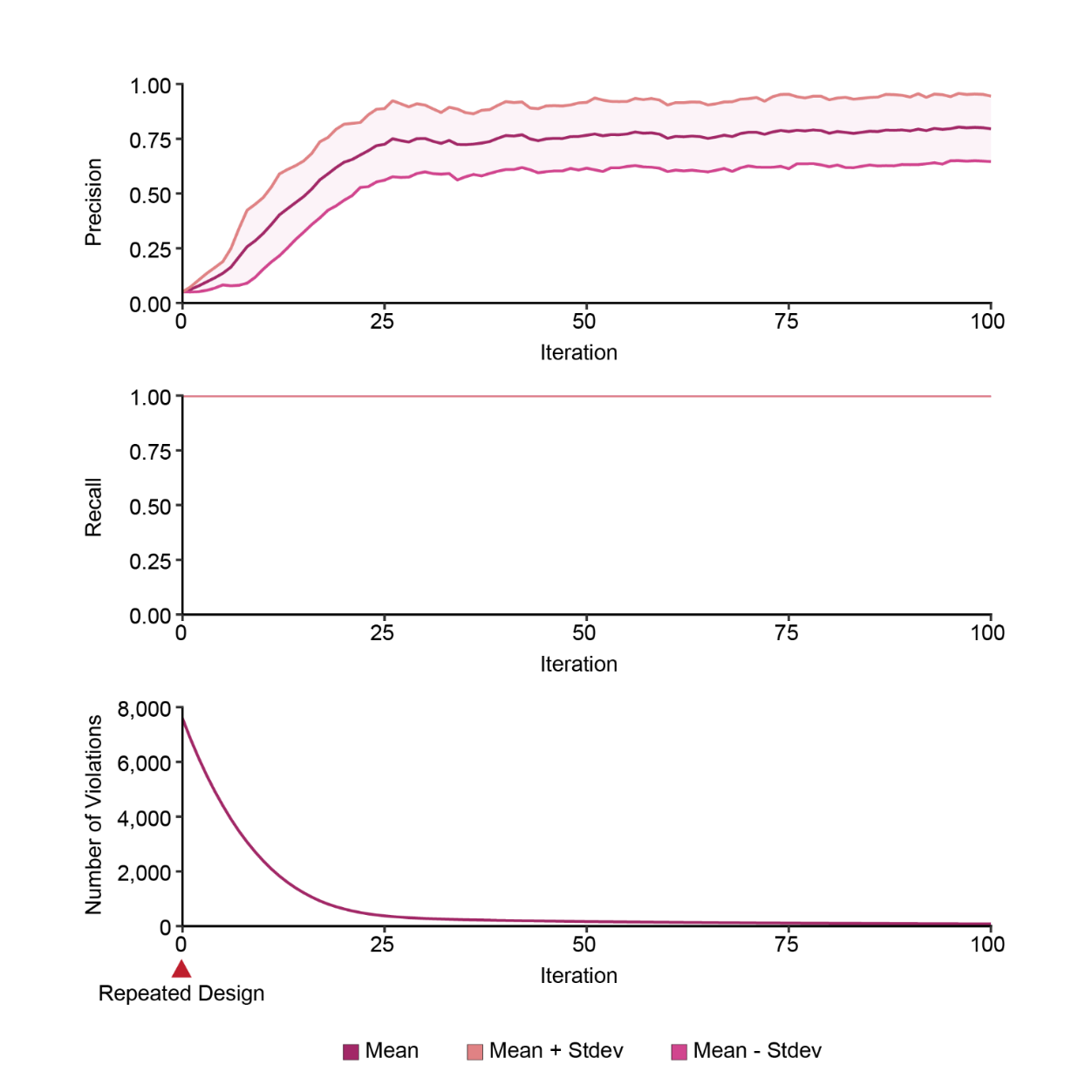


**Figure S1.** Characterization of the assumption behind minimizing co-occurrence violations in non-overlapping designs. ACE design generation (without epitope similarity prediction) was simulated 100 times, yielding 100 pseudo-trajectories. Each simulation consisted of optimizing a repeated design over 100 iterations resulting in as many separate designs per simulation. Both empirical and expectation maximization deconvolution was performed for each simulation iteration. The metrics were reported on deconvolved peptides where a positive peptide is defined as appearing in 3 positive pools with non-zero estimated spots. The top panel shows the average precision along with the standard deviation. The middle panel shows the average recall (sensitivity). The bottom panel shows the number of peptide pair co-occurrence violations. These metrics were captured at each optimization iteration across 100 replicates. The design parameters were 400 peptides pooled into 20 peptides per pool with 3x coverage at a 1% positivity rate. The peptide spot counts were simulated without stochastic effects.


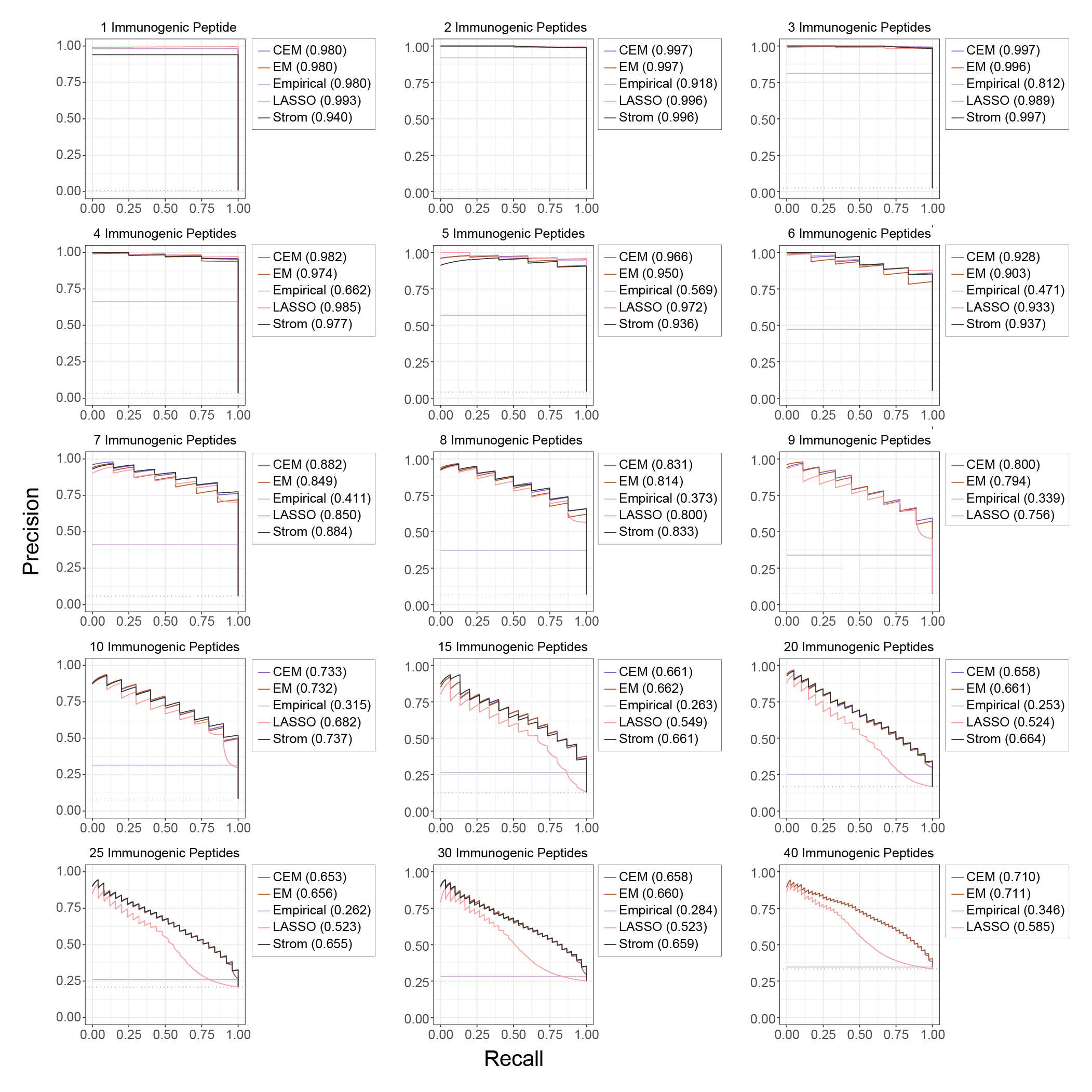


**Figure S2.** Comparison of deconvolution area under the precision-recall curves between ACE and Strom for 120 peptides. The plots show the average AUPRC from 100 simulation runs (CEM = constrained expectation maximization, EM = expectation maximization). For a fair comparison of the deconvolution methods, the same design configurations (generated with ACE sequence similarity prediction) were used for all deconvolution methods in each simulation run. The AUPRC values for Strom’s deconvolution, available as a Shiny app, are not plotted for 9 and 40 immunogenic peptide datasets because it failed to run at least 20 (20%) out of 100 runs.


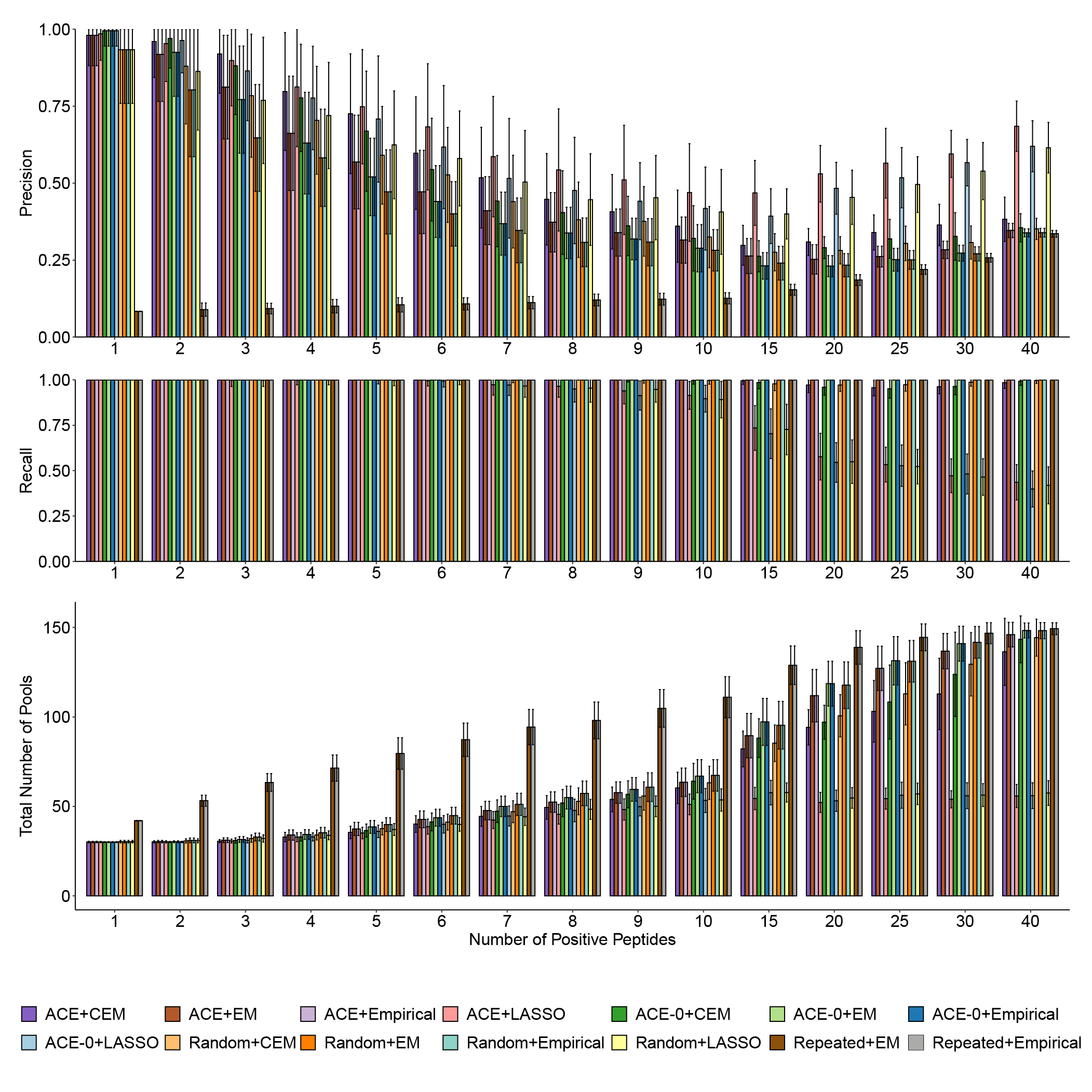


**Figure S3.** Simulated benchmark study evaluation on 120 peptides for different combinations of design configuration and deconvolution strategies. Spot counts for 120 peptides were simulated for different numbers of positive peptides with 12 peptides per pool and 3 technical replicates (i.e. 3x coverage). The top panel shows the average precision, the middle panel show the average recall, and the bottom panel shows the average total number of pools (sum of first round and confirmatory second round assay pools) across 100 replicates for each method. The error bars show $\pm1$ standard deviation.

**
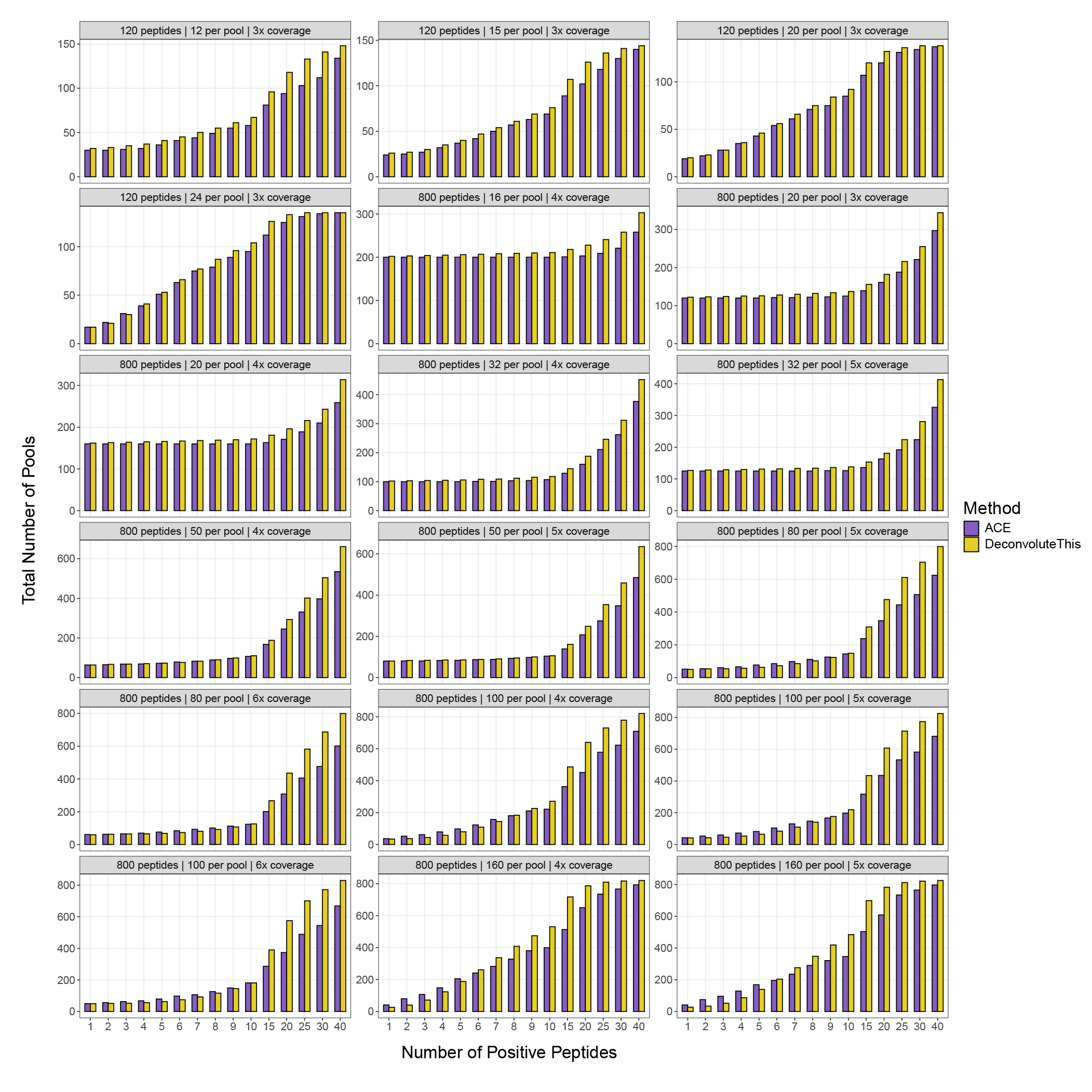
**

**Figure S4.** Comparison of ACE and DeconvoluteThis by the total number of pools. DeconvoluteThis design configurations with at least 3x coverage found in the original DeconvoluteThis paper were created using ACE (sequence-aware design generation followed by CEM deconvolution). For each number of positive peptides, 100 simulations were run using ACE without stochastic effects and the average total number of pools is reported. The total number of pools for DeconvoluteThis was taken from the original manuscript.

**
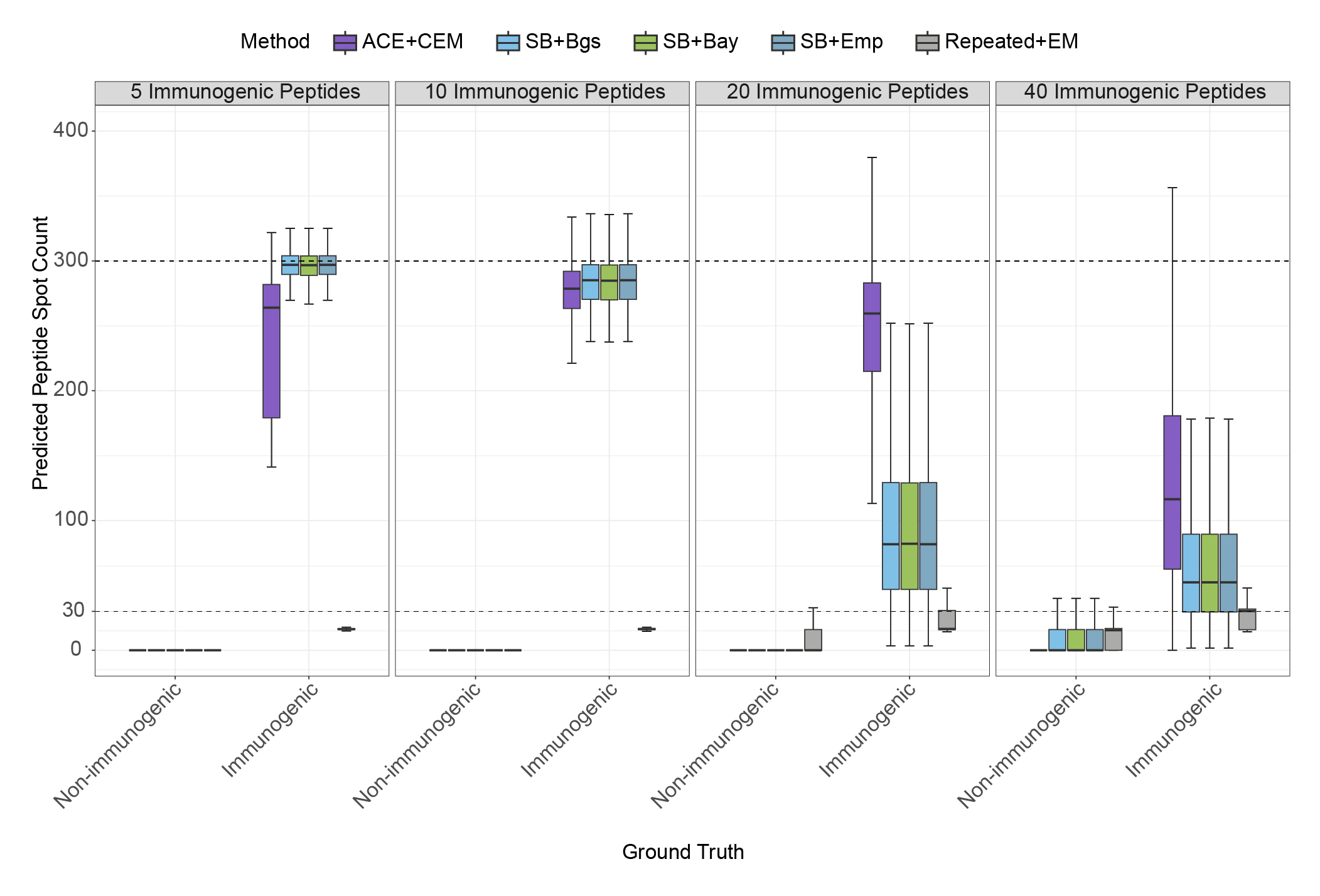
**

**Figure S5.** Comparison of predicted peptide spots for 800 peptides between ACE and Strandberg deconvolution methods. Peptides were sampled from the IEDB data held-out from ACE neural engine training with spot counts sampled from a Poisson distribution ($\lambda_{immunogenic}=300, \lambda_{non-immunogenic}=30$), shown above by the two dotted lines. For each number of immunogenic peptides, 100 simulations were run. There is a general trend where the predicted peptide spots converge as the positivity rate increases across all 5 methods. The ACE design generation paired with constrained EM deconvolution method was more robust to variations in positivity rates compared to Strandberg’s (SB) deconvolution methods (emp = empirical positivity selection, bay = Bayesian Predictive ELISpot Criterion; BPEC, bgs = background subtracted) when 20 and 40 peptides were immunogenic out of 800 peptides.


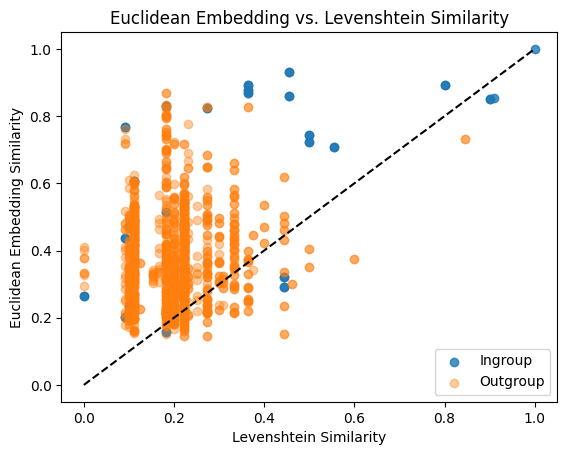


**Figure S6.** Correlation plot between Euclidean and Levenshtein similarity. Euclidean distance captures the high sequence similarity epitopes and some of the low sequence similarity ingroup epitopes while increasing the Euclidean similarity between outgroup pairs.


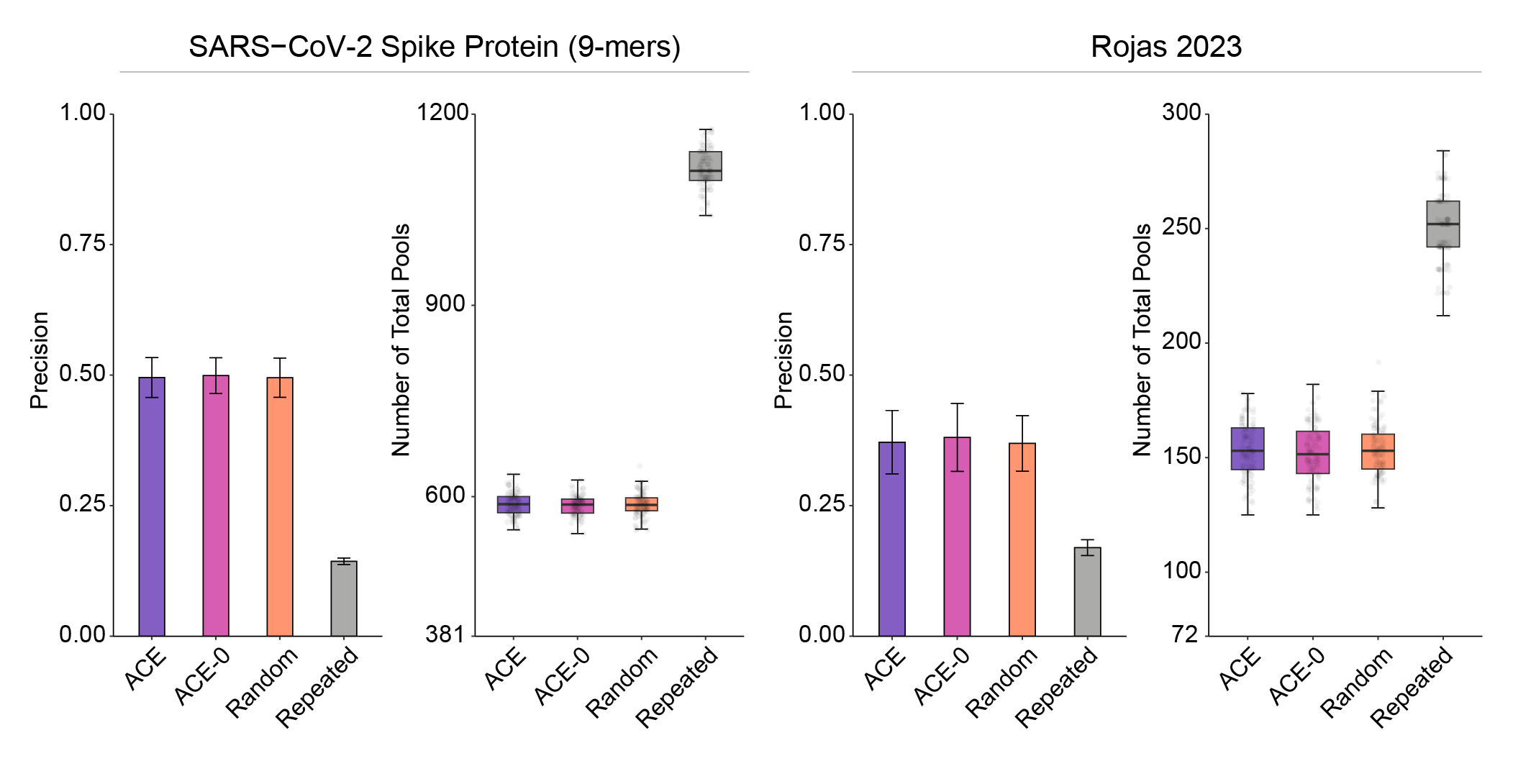


**Figure S7.** Assay performance on 1,265 SARS-CoV-2 spike protein 9-mer sequences and 232 candidate neoantigens from a pancreatic cancer cohort (Rojas et al., Nature 2023). A sliding window of size $k=9$ was applied over the 1,273 SARS-CoV-2 spike protein amino acids. The immunogenicity labels were taken from the original manuscript for the pancreatic cancer neoantigens. The precision and total number of pools are reported for 100 simulations. The error bars represent $\pm1$ standard deviation.

**
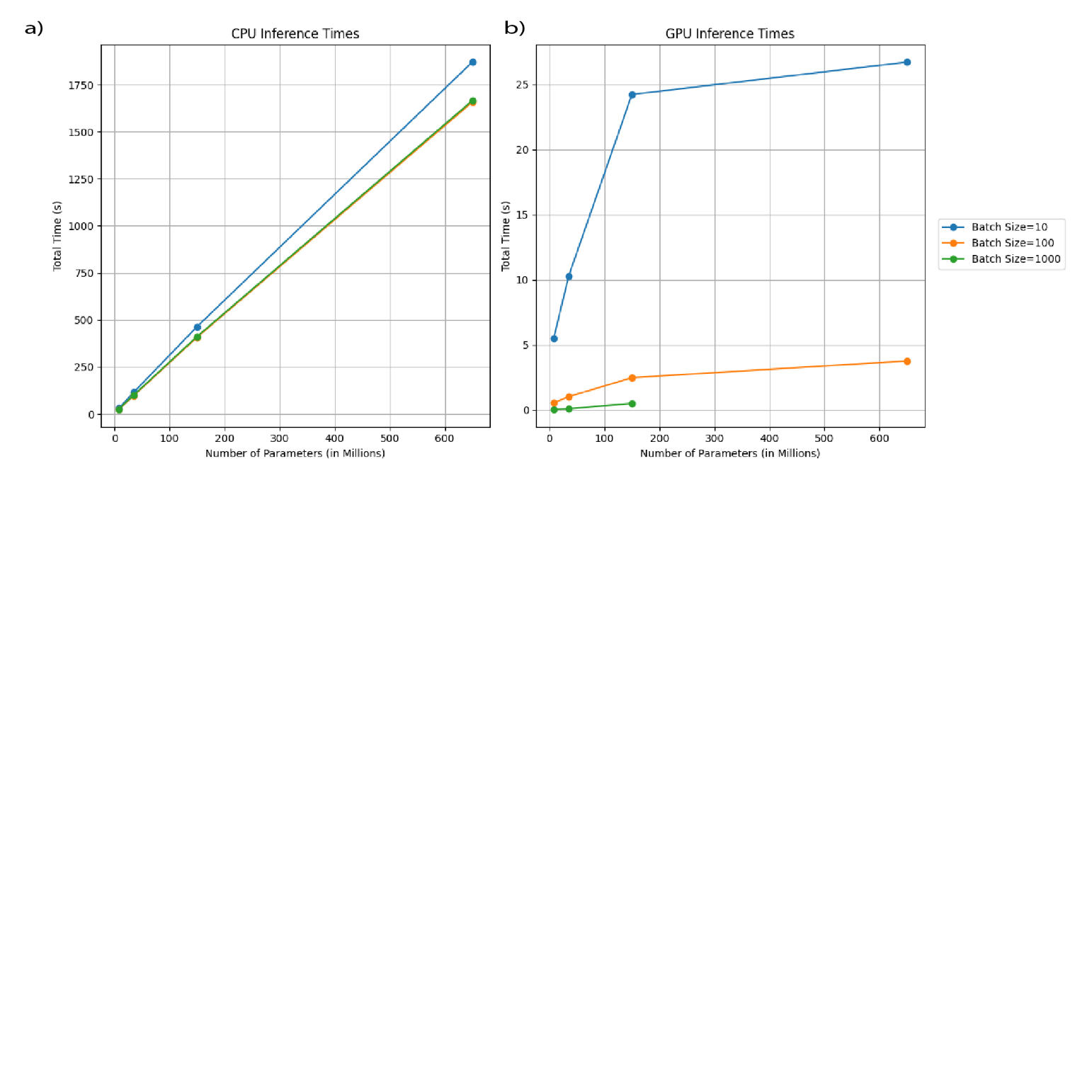
**

**Figure S8.** Speed performance benchmark on 10,000 randomly generated 9-mer sequences. (a) Total inference time on CPU as a function of parameter count. (b) Total inference time on GPU as a function of parameter count. The 650M inference on GPU for a batch size of 1,000 is not shown due to an out-of-memory error.
