# Supplementary material for: ACE configurator for ELISpot: optimizing combinatorial design of pooled ELISpot assays with an epitope similarity model
Source: Brief Bioinform. 2024 Jan 4;25(1):bbad495. doi: 10.1093/bib/bbad495 (PMC10768796; doi:10.1093/bib/bbad495)
Supplement: ACE_Supplementary_Notes_v3_bbad495 [file ace_supplementary_notes_v3_bbad495.docx]

SUPPLEMENTARY NOTES

**ACE Configurator for ELISpot (ACE): Optimizing Combinatorial
Design of Pooled ELISpot Assays with an Epitope Similarity Model**

Jin Seok Lee^1,4,5,*^, Dhuvarakesh Karthikeyan^1,4,5,*^, Misha Fini^1,3^,
Benjamin G. Vincent^1,2,3,4,5^, Alex Rubinsteyn^1,4,5,6^

^1^Lineberger Comprehensive Cancer Center, University of North Carolina at Chapel Hill, Chapel Hill, NC

^2^Division of Hematology, Department of Medicine, University of North Carolina at Chapel Hill, Chapel Hill, NC

^3^Department of Microbiology and Immunology, UNC School of Medicine, Chapel Hill, NC, USA

^4^Computational Medicine Program, UNC School of Medicine, Chapel Hill, NC, USA

^5^Curriculum in Bioinformatics and Computational Biology, UNC School of Medicine, Chapel Hill, NC, USA

^6^Department of Genetics, University of North Carolina at Chapel Hill, Chapel Hill, NC 27599 USA

*Contributed equally, co-first authors.

# Table of Contents

**Supplementary Note 1.** Immunogenicity and Pooled ELISpot Assay Designs.

**Supplementary Note 2.** ACE Neural Engine.

# Supplementary Note 1. Immunogenicity and Pooled ELISpot Assay Designs


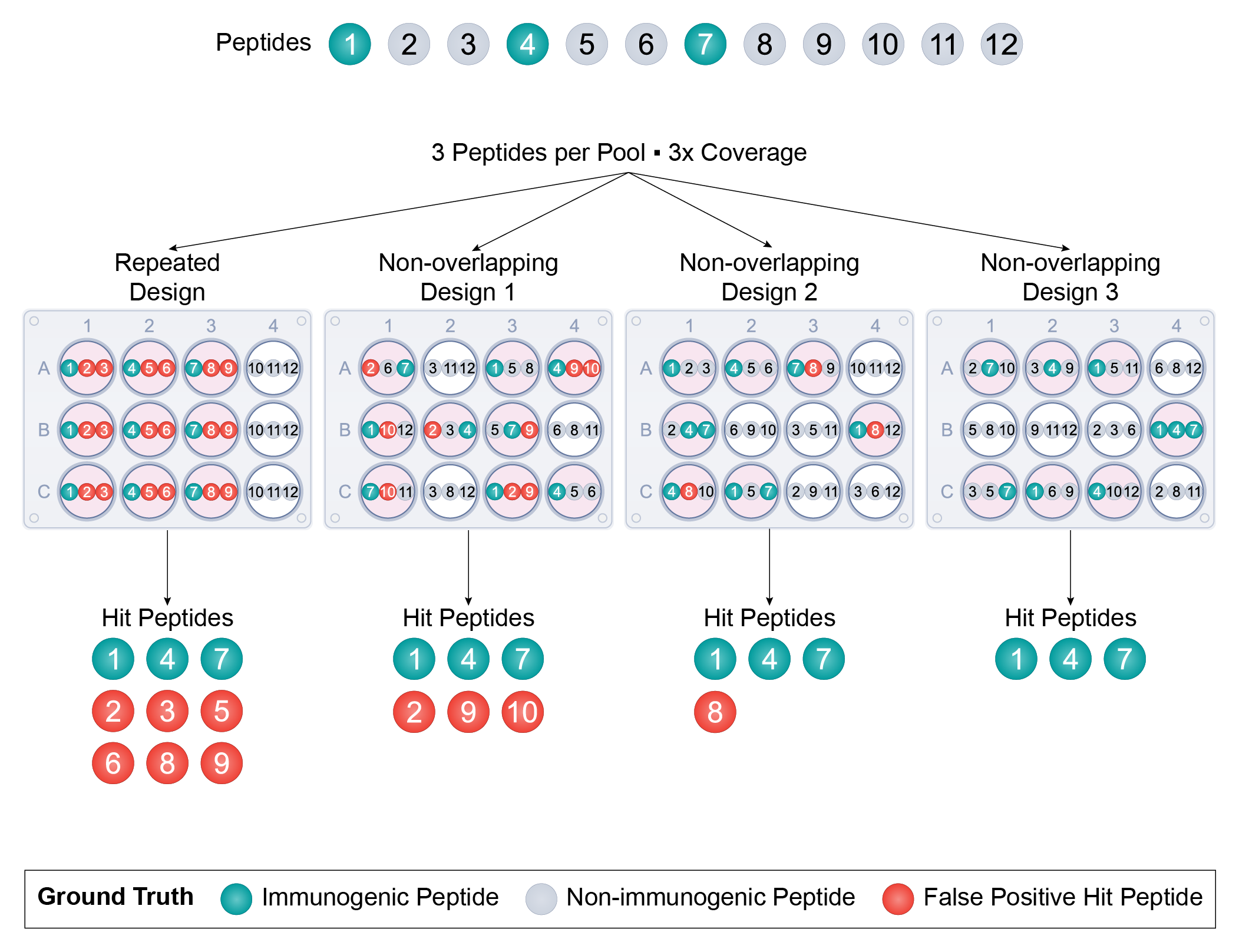


**Figure 1.** Variability in precision across equivalent Non-Overlapping Designs (NODs).

In the current paradigm of pooled ELISpot setup, non-overlapping designs (NODs) that minimize the number of peptide co-occurrences across replicates are considered optimal [1]. However, when the number of true positive peptides matches or exceeds the number of technical replicates, false positives may arise due to non-immunogenic peptides coinciding with different immunogenic ones. Various NODs that meet this constraint exist. We hypothesized that these equivalent classes could vary in experimental efficiency.

We illustrate how the different placements of true positive peptides affect the number of predicted positives with an example of 12 peptides assigned into pools of 3 peptides across 3 technical replicates (also referred to as $coverage=3$) (Figure 1). We first show the repeated block design, commonly used in practice, to baseline against the NODs. The repeated block design segments the set of peptides into disjoint groups and repeats these groups as technical replicates. This pooling approach, however, makes it difficult to determine which specific peptides are immunogenic since there is no variation in the composition of repeated groups. An empirical deconvolution of the repeated block design identifies the peptides 1, 4, 7 as well as 2, 3, 5, 6, 8, and 9 as hits (precision = 0.333). We then consider three NODs with varying degrees of immunogenic peptide pooling. NOD 1 prevents mixing of the true positive peptides and results in a precision of 0.500. NOD 2 pairs 4 and 7 as well as 1 and 7, resulting in a precision of 0.750. NOD 3 combines all 3 true positives for a precision of 1.000. Notably, in separating the immunogenic peptides, NOD 1 has the unintended consequence of maximizing the number of positive wells, thus increasing the chances that a non-immunogenic peptide shares a pool with different immunogenic epitopes across coverages. NODs in ELISpot assays offer a starting point; peptides’ characteristics can optimize hit assignment and deconvolution.

To this end, we reasoned that NODs could yield highest precision when immunogenicity can be accurately predicted. However, in practice, immunogenicity predictors generalize poorly on unseen epitopes and have been unable to outperform MHC binding predictors [2]. ACE approximates immunogenicity by pooling similar epitopes predicted to bind common T-cell receptors, exploiting ELISpot wells as samples (without replacement) of donor T-cell repertoires.

# Supplementary Note 2. ACE Neural Engine


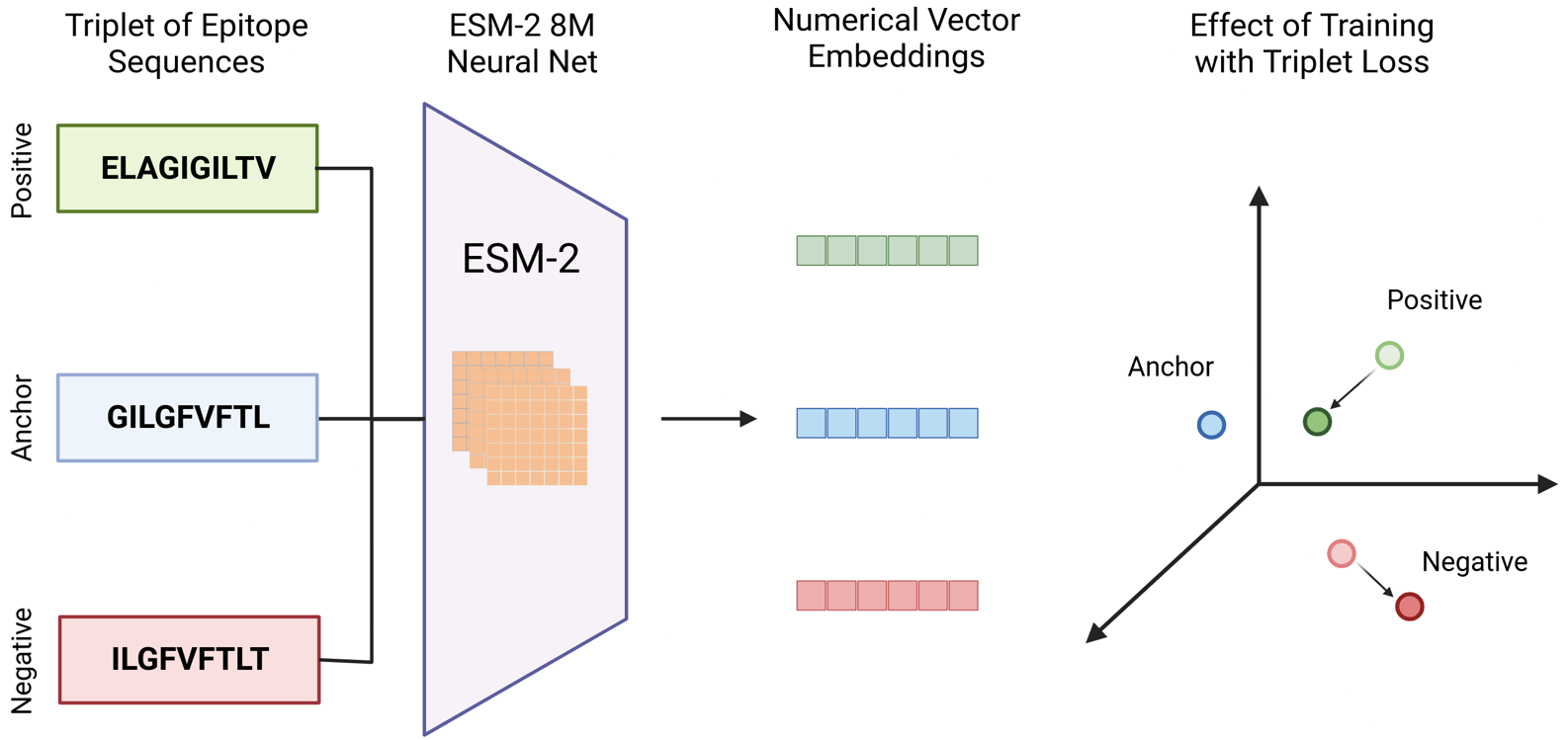


**Figure 2.** Learning Protein Embeddings via Triplet Loss.

**Modeling T-cell Receptor Reactivity via Learned Sequence Representations**

Predicted T-cell receptor (TCR) reactivity is factored into ACE by the fine-tuned embeddings for epitope sequences. Specifically, the parameters of the pre-trained ESM-2 model are adjusted through backpropagation of a loss computed on a sequence triplet comprised of an anchor sequence, a positive sequence (one that shares at least one cognate TCR with the anchor), and a negative sequence (one that shares non-cognate TCRs with the anchor). Loss is calculated based on the difference between the anchor::positive and anchor::negative embeddings’ Euclidean distances. A margin is enforced so that trivial embeddings are not learned. Encoding TCR specificity is achieved by iteratively updating the weights as described above such that the learned embeddings of epitopes that share TCRs are brought closer than the embeddings of epitopes that share no TCR over training epochs.

The above learning paradigm has been shown to work well in the few-shot regime where training examples per class are scarce, such as facial recognition training data [3]. By this approach, absolute TCR reactivity (i.e. cognate receptor sequence prediction) cannot be inferred from this model directly. This framework is useful for injecting the relative structure of the TCR-epitope cross reactivity network into the model embeddings’ latent space.

**Data Provenance**

Paired TCR:pMHC data was taken from the Immune Epitope Database (IEDB). Due to the constraint of requiring paired TCR data to serve as context, the data were positive-only. From this, all sequences derived from SARS-CoV-2, SARS-CoV-1, and other coronaviruses were removed. We also explicitly filtered out 37 epitopes that were assayed by Cameron et al. (2013) to ensure a proper holdout validation set (Main Figure 4f-g). The data were split such that no epitopes in the training data appeared in the validation data. The data were also weighted by MHC-I allele to ensure that a roughly equal representation of sequence biases from differential abundance of MHC alleles was captured in both the training and validation data. This resulted in a total of 520 unique epitopes which were split into a training size of 453 epitopes and test size of 67 (~12%).

**Triplet Loss and Mining Strategy**

$$\mathcal{L(}\boldsymbol{A,P,N)=max}\left( \left\| \boldsymbol{f(A)-f(P)} \right\|_{\boldsymbol{2}}\boldsymbol{-}\left\| \boldsymbol{f(A)-f(N)} \right\|_{\boldsymbol{2}}\boldsymbol{+}\alpha\boldsymbol{,0} \right)$$

The triplet loss [3] is calculated using three separate data points: the anchor, a positive example, and a negative example. The anchor is the query example from the data which serves as the reference by which to choose a positive example (instance from the same class as the anchor) and negative example (instance from another class). In our case, we set the anchor as the query epitope sequence, the positive example is an epitope that shares a TCR with the query epitope and the negative example is a sampled epitope that shares no TCR with the anchor. Each sequence is passed through the model yielding f(A), f(P), and f(N) and the loss is calculated as the difference in the Euclidean distances between the anchor::positive and the anchor::negative embeddings. The $\alpha$ term is the desired margin between the differences in the class boundaries. In our study we set this value to be 1.0. The model training progresses as non-trivial or hard negatives are chosen where the distance between the anchor and negative examples is less than the distance between the anchor and positive sequence, a process known as triplet mining. For our purposes, hard triplets were mined by sorting the negative peptides based on increasing Levenshtein edit distance and sampled during training with a 35% chance of choosing the negative with the closest Levenshtein distance and sampling equally from the closest 20 negatives. A similar scheme was used for positive mining, based on sequences that were sorted by the degree of shared TCRs with other epitopes and sub-sorted based on Levenshtein edit distance. The model was trained for 24,000 iterations with batch size of 64, where batches were balanced by epitopes such that for a single epoch each epitope was used as an anchor only once with the positive and negative mining methods mentioned above.

**Model Size and Training History**


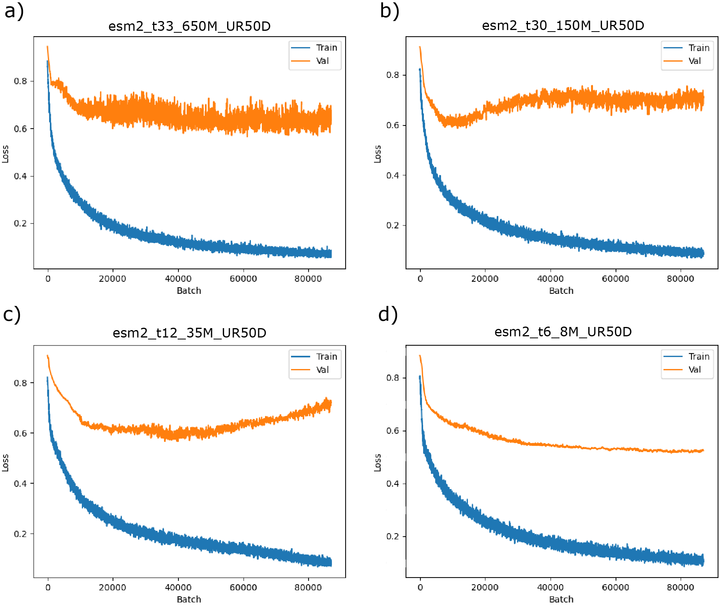


**Figure 3.** Model training curves from different model checkpoints. Each model was fine-tuned using a learning rate of 1e-6, compared to the learning rate used in pretraining (4e-4) with the Adam optimizer. Training history is reported with the loss logged every 10 iterations at batch size of 64.


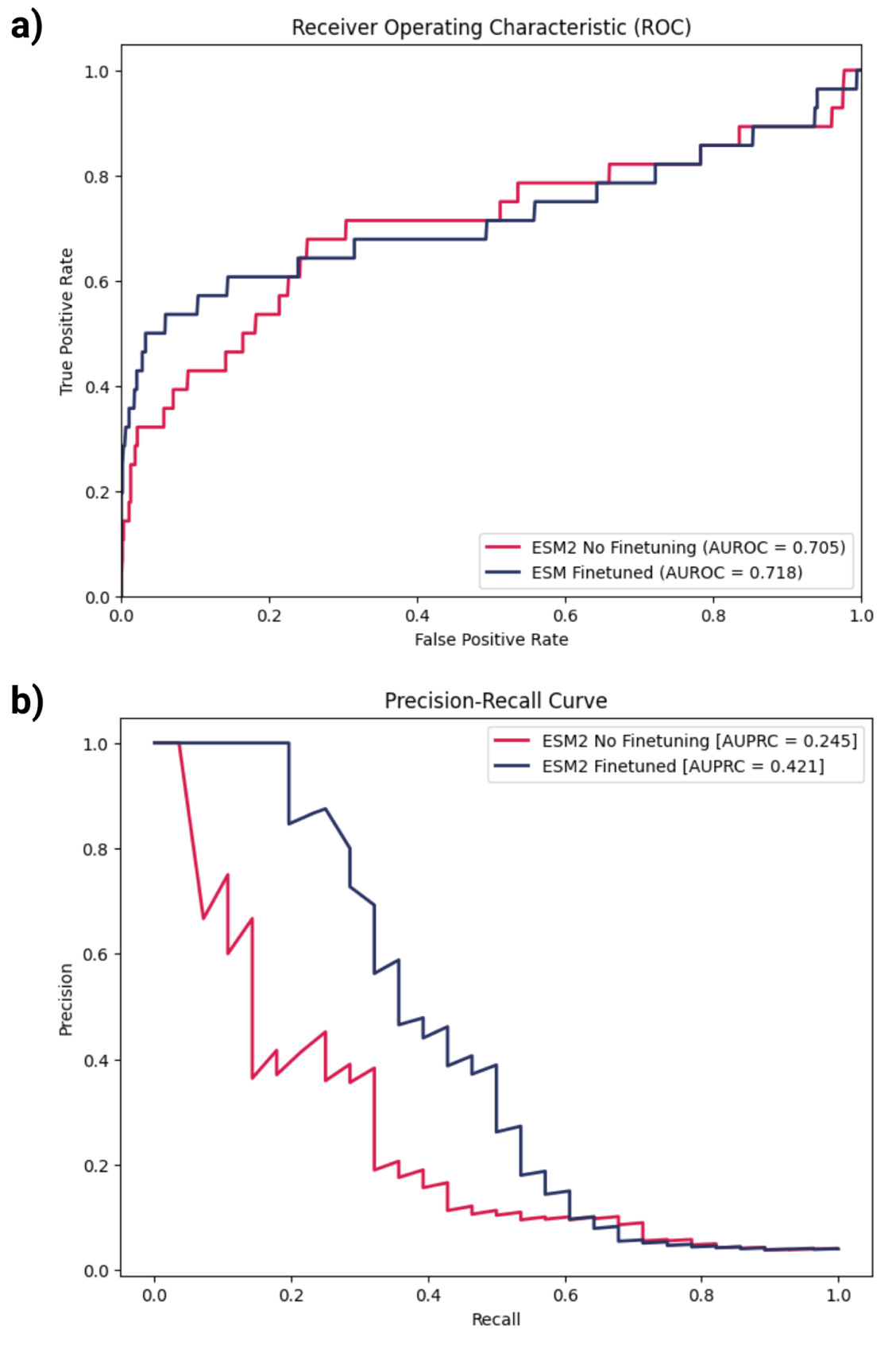


**Figure 4.** ESM-2 8M model performance before and after fine-tuning. Pairwise Euclidean distances computed for held-out in-group and out-group pairs and assigned labels. **(a)** Area under the receiver operating characteristic curve (AUROC) of the models before and after fine-tuning. **(b)** Area under the precision recall curve (AUPRC) of the models before and after fine-tuning.

# REFERENCES

1. Roederer M, Koup RA: **Optimized determination of T cell epitope responses.** *J Immunol Methods* 2003, **274:**221-228.

2. Buckley PR, Lee CH, Ma R, Woodhouse I, Woo J, Tsvetkov VO, Shcherbinin DS, Antanaviciute A, Shughay M, Rei M, et al: **Evaluating performance of existing computational models in predicting CD8+ T cell pathogenic epitopes and cancer neoantigens.** *Brief Bioinform* 2022, **23**.

3. Schroff F, Kalenichenko D, Philbin J: **FaceNet: A unified embedding for face recognition and clustering.** In *2015 IEEE Conference on Computer Vision and Pattern Recognition (CVPR)*; *7-12 June 2015*. 2015: 815-823.
